# The safety and efficacy of first-line atezolizumab plus bevacizumab in patients with unresectable hepatocellular carcinoma: A multicenter real-world study from Turkey

**DOI:** 10.1097/MD.0000000000035950

**Published:** 2023-11-10

**Authors:** Arif Akyildiz, Deniz Can Guven, Ahmet Anil Ozluk, Rashad Ismayilov, Emel Mutlu, Olcun Umit Unal, Ibrahim Yildiz, Yakup Iriagac, Serdar Turhal, Sinem Akbas, Ertugrul Bayram, Tugba Akin Telli, Fatma Paksoy Turkoz, Melike Ozcelik, Mert Erciyestepe, Oguzhan Selvi, Burcu Gulbagci, Ismail Erturk, Zehra Sucuoglu Isleyen, Seda Kahraman, Mutianur Ozkorkmaz Akdag, Buket Hamitoglu, Ilkay Tugba Unek, Caglar Unal, İlhan Hacibekiroglu, Cagatay Arslan, Abdulmunir Azizy, Kaan Helvaci, Umut Demirci, Omer Dizdar, Mert Basaran, Erdem Goker, Mehmet Ali Sendur, Suayib Yalcin

**Affiliations:** a Department of Medical Oncology, Hacettepe University Cancer Institute, Ankara, Turkey; b Department of Medical Oncology, Ege University Faculty of Medicine, Izmir, Turkey; c Department of Internal Medicine, Hacettepe University Faculty of Medicine, Ankara, Turkey; d Department of Medical Oncology, Erciyes University Faculty of Medicine, Kayseri, Turkey; e Department of Medical Oncology, Bozyaka Education and Research Hospital, Izmir, Turkey; f Department of Medical Oncology, Acibadem University Hospital, Istanbul, Turkey; g Department of Medical Oncology, Namik Kemal University, Tekirdag, Turkey; h Department of Medical Oncology, Anadolu Medical Center, Kocaeli, Turkey; i Department of Medical Oncology, Koc University Hospital, Istanbul, Turkey; j Department of Medical Oncology, Cukurova University Faculty of Medicine, Adana, Turkey; k Department of Medical Oncology, Marmara University School of Medicine, Istanbul, Turkey; l Department of Medical Oncology, Istinye University, Medical Park Goztepe Hospital, Istanbul, Turkey; m Department of Oncology, Kartal Lutfi Kirdar Teaching and Research Hospital, Istanbul, Turkey; n Department of Medical Oncology, Prof Dr. Cemil Tascioglu City Hospital, Istanbul, Turkey; o Department of Medical Oncology, Sakarya University Faculty of Medicine, Sakarya, Turkey; p Department of Medical Oncology, Gulhane School of Medicine, Ankara, Turkey; q Department of Medical Oncology, Faculty of Medicine, Bezmialem Vakif University, Istanbul, Turkey; r Department of Medical Oncology, Ankara City Hospital, Yildirim Beyazit University, Ankara, Turkey; s Department of Medical Oncology, Kocaeli University Faculty of Medicine, Kocaeli, Turkey; t Department of Medical Oncology, Dokuz Eylül University Faculty of Medicine, Izmir, Turkey; u Department of Medical Oncology, Gayrettepe Florence Nightingale Hospital, Istanbul, Turkey; v Department of Medical Oncology, Izmir University of Economics Faculty of Medicine, Izmir, Turkey; w Department of Medical Oncology, Istanbul University Institute of Oncology, Istanbul, Turkey; x Department of Medical Oncology, Memorial Ankara Hospital, University of Uskudar, Ankara, Turkey.

**Keywords:** atezolizumab, bevacizumab, hepatocellular carcinoma, immunotherapy

## Abstract

The aim of the study was to evaluate the real-world clinical outcomes of atezolizumab and bevacizumab (Atez/Bev) as the initial therapy for advanced hepatocellular carcinoma (HCC). We retrospectively analyzed 65 patients treated with Atez/Bev for advanced HCC from 22 institutions in Turkey between September 2020 and March 2023. Responses were evaluated by RECIST v1.1 criteria. The median progression-free survival (PFS) and overall survival (OS) were calculated using the Kaplan–Meier method. Cox regression model was employed to conduct multivariate analyses. The median age was 65 (range, 22–89) years, and 83.1% of the patients were male. A total of 1.5% achieved a complete response, 35.4% had a partial response, 36.9% had stable disease, and 26.2% had progressive disease. The disease control rate was 73.8% and associated with alpha-fetoprotein levels at diagnosis and concomitant antibiotic use. The incidence rates of any grade and grade ≥ 3 adverse events were 29.2% and 10.7%, respectively. At a median follow-up of 11.3 (3.4–33.3) months, the median PFS and OS were 5.1 (95% CI: 3–7.3) and 18.1 (95% CI: 6.2–29.9) months, respectively. In univariate analyses, ECOG-PS ≥ 1 (relative to 0), Child-Pugh class B (relative to A), neutrophil-to-lymphocyte ratio (NLR) > 2.9 (relative to ≤ 2.9), and concomitant antibiotic use significantly increased the overall risk of mortality. Multivariate analysis revealed that ECOG-PS ≥ 1 (HR: 2.69, *P* = .02), NLR > 2.9 (HR: 2.94, *P* = .017), and concomitant antibiotic use (HR: 4.18, *P* = .003) were independent predictors of OS. Atez/Bev is an effective and safe first-line therapy for advanced-stage HCC in a real-world setting. The survival benefit was especially promising in patients with a ECOG-PS score of 0, Child-Pugh class A, lower NLR, and patients who were not exposed to antibiotics during the treatment.

## 1. Introduction

Hepatocellular carcinoma (HCC) is a highly prevalent cancer and a major cause of cancer-related mortality, ranking as the sixth most common malignancy globally.^[1–3]^ The incidence of HCC has been steadily increasing over the past few decades.^[4]^ It is mostly caused by chronic hepatitis B virus (HBV) or hepatitis C virus (HCV) infection, alcohol abuse, or nonalcoholic fatty liver disease.^[5]^ While the curative treatments such as surgery, liver transplantation, and ablation could be effective in the early stages, a significant number of patients present with advanced, unresectable HCC, which is associated with a poor prognosis.^[6]^ In order to tackle this challenge, diverse systemic treatment options have been developed, which encompass targeted therapies and immunotherapies.^[7]^ However, due to the complex pathogenesis and heterogeneous etiology of HCC, effective treatment strategies remain a considerable challenge.

Sorafenib, the first targeted therapy, demonstrated a survival benefit in advanced HCC and has been the standard of care for almost a decade.^[8]^ However, it has limitations in terms of efficacy and tolerability, highlighting the need for additional treatment options. Lenvatinib, another multikinase inhibitor, has shown non-inferiority to sorafenib in the phase III REFLECT trial conducted in 2018.^[9]^ Besides multikinase inhibitors, immunotherapy has gained attention as a potential treatment approach for advanced HCC.^[10,11]^ In 2020, atezolizumab, an anti-programmed death ligand 1, monoclonal antibody, and bevacizumab, an anti- vascular endothelial growth factor monoclonal antibody, were approved as first-line therapy for unresectable or metastatic HCC, changing the treatment landscape for advanced HCC.^[12]^ The phase III IMbrave150 study demonstrated that the combination of atezolizumab and bevacizumab (Atez/Bev) improved overall survival (OS) and progression-free survival (PFS) compared to sorafenib.^[12]^ However, the utilization of these immunotherapies comes with certain challenges, including the potential for immune-related adverse events and the necessity for biomarkers to predict treatment response. Clinical outcomes of the combined use of Atez/Bev in routine clinical practice have been described in only a small number of well-designed multicenter studies. In this multicenter study, we aimed to assess the clinical effectiveness and safety of Atez/Bev therapy as first-line regimen for the treatment of patients with unresectable HCC in a real-world setting.

## 2. Materials and methods

### 2.1. Patients characteristics

As part of routine clinical care, a retrospective multicenter study was undertaken to assess the outcomes of patients with unresectable or metastatic HCC who underwent treatment with a combination of atezolizumab and bevacizumab. The study included patients from 22 different institutions, spanning the period from September 2020 to March 2023. This study included ≥ 18 years old patients who fulfilled the criteria for advanced HCC based on the Barcelona Clinic Liver Cancer (BCLC) guidelines. Additionally, inclusion criteria required the presence of histological or radiological evidence of HCC, in accordance with the criteria established by either the American Association for the Study of Liver Diseases or the European Association for the Study of the Liver.^[13,14]^ The study protocol was approved by Hacettepe University Ethics Board and Turkish Medicines and Medical Devices Agency.

### 2.2. Treatment characteristics and efficacy

All enrolled patients received intravenous administration of atezolizumab at a dosage of 1200 mg and bevacizumab at a dosage of 15 mg/kg every 3 weeks. Treatment was continued until disease progression or the development of intolerable toxicity. Dose adjustments and treatment interruptions were permitted during the study, based on the severity of drug-related toxicity. Baseline assessments were conducted prior to the initiation of treatment, and subsequent liver dynamic computed tomography or magnetic resonance imaging scans were performed at intervals of 8 to 12 weeks following the administration of atezolizumab in combination with bevacizumab. Subsequent imaging was conducted every 8 to 12 weeks thereafter. The Response Evaluation Criteria in Solid Tumors was employed to assess the treatment response. This criterion categorizes the response into 4 groups: complete response (CR), partial response (PR), stable disease (SD), and progressive disease.^[15]^ The disease control rate (DCR) was calculated as the percentage of patients who achieved CR, PR, or SD. The objective response rate (ORR) was calculated as the sum of the CR and PR rates. Alpha-fetoprotein (AFP) levels were assessed at baseline and subsequently with each magnetic resonance imaging or computed tomography scans. Safety assessments were performed using the National Cancer Institute’s Common Terminology Criteria version 5.0.

### 2.3. Statistical analysis

Descriptive statistics were presented as frequency (percent) or median (range, min–max). The χ^2^ or Fisher’s exact tests were used to compare the proportions in different categorical groups. Continuous variables were analyzed with the Mann–Whitney *U* test. To evaluate the prognostic significance of the neutrophil-to-lymphocyte ratio (NLR) for mortality, receiver operating characteristic curve analysis was conducted. OS was calculated from the initiation of atezolizumab plus bevacizumab treatment until death from any cause. PFS was defined as the time from the initiation of the treatment to radiological tumor progression or death from any cause. Survival estimates were calculated with the Kaplan–Meier method. The log-rank test was used to identify the independent effects on OS. Multivariate analyses were performed using Cox regression. An overall type-1 error level was used to infer statistical significance.

## 3. Results

### 3.1. Baseline characteristics

A total of 65 patients [54 (83.1%) men] included in the study had a median age of 65 (22–89) years, and 31 (47.7%) were ≥ 65 years of age. Thirty-eight (58.5%) patients had an Eastern Cooperative Oncology Group-Performance Status (ECOG-PS) of 0, while 26 (40%) had a score of 1, and 1 (1.5%) had a score of 2. Hypertension was the most common comorbidity (29.2%), followed by diabetes mellitus (23%). Regarding the Child-Pugh classification, 58 (89.2%) patients had class A disease, and 7 (10.8%) had class B disease. At the initiation of Atez/Bev therapy, the distribution of BCLC stages for the HCC patients were as follows: 1 (1.5%) patient with stage A, 32 (49.2%) patients with stage B, and 32 (49.2%) patients with stage C. The most commonly observed underlying liver diseases were chronic viral hepatitis, either secondary to HBV (n = 24, 36.9%) or HCV infection (n = 6, 9.2%). HCC was associated with nonalcoholic steatohepatitis in 10 (15.3%) patients, alcohol in 3 (4.6%) patients, and other causes including smoking, environmental toxins, diabetes mellitus, and unknown etiologies in 22 (33.8%) patients. For chronic HBV, patients received entecavir (n = 18) or tenofovir (n = 6); 6 patients with HCV received a combination of glecaprevir and pibrentasvir. Among the study patients, 22 (33.9%) had received at least one prior locoregional or radical treatment, with transarterial chemoembolization being the most common prior therapy (18.5%). Cirrhosis, either clinically or radiologically diagnosed, was present in approximately 41.5% of the patients. The median serum AFP level at baseline was 80 (1.4–409,220) ng/mL. Upon baseline assessment, 36 patients (55.4%) exhibited extrahepatic spread. Concurrent with Atez/Bev, 2 and 5 patients required intravenous and peroral antibiotics, respectively. Three patients received beta-lactam, 2 patients received quinolones, and 2 patients received combined beta-lactam and macrolide antibiotics. The baseline characteristics of the patients are summarized in Table 1.

**Table 1:** Baseline characteristics of patients (65 patients).

Characteristics		n	%
Age, median (range), years		65 (22–89)	
Sex	Women	11	16.9
	Men	54	83.1
ECOG-PS	0	38	58.5
	1	26	40.0
	2	1	1.5
Child-Pugh Score	5	47	72.3
	6	11	16.9
	7	5	7.7
	8	2	3.1
BCLC Stage	A	1	1.5
	B	32	49.2
	C	32	49.2
Etiology	Hepatitis B	24	36.9
	Hepatitis C	6	9.2
	NASH	10	15.3
	Alcohol	3	4.6
	Others	22	33.8
Cirrhosis	Presence	27	41.5
	Absence	38	58.5
Comorbidities	Hypertension	19	29.2
	Diabetes	15	23
	CAD	6	9.2
	Others	13	20
	Absence	38	58.5
Extrahepatic metastasis	Lung	18	27.7
	Bone	10	15.4
	Others	8	12.3
Prior therapy	TACE	12	18.5
	TARE	10	15.4
Histopathology	Presence	51	78.5
	Absence	14	21.5
Antibiotic use	User	7	10.7
	Non-user	58	89.3
AFP, median (range), ng/mL		80 (1.4–409220)	
Platelets, median (range), 10^3^/μL		221 (46–481)	
Total bilirubin, median (range), mg/dL		0.89 (0.19–4)	
Albumin, median (range), g/dL		3.96 (2.3–4.9)	
INR, median (range)		1.08 (0.8–1.7)	

AFP = alpha-fetoprotein, BCLC = Barcelona Clinic Liver Cancer, CAD = coronary artery disease, ECOG-PS = Eastern Cooperative Oncology Group-Performance Status, INR = international normalized ratio, NASH = nonalcoholic steatohepatitis, TACE = transarterial chemoembolization, TARE = transarterial radioembolization.

### 3.2. Treatment responses

Overall, the treatment yielded a CR in 1 (1.5%) case, PR in 23 (35.4%) cases, SD in 24 (36.9%) cases, and progressive disease in 17 (26.2%) cases. ORR was 36.9%, and the DCR was 73.8% (Table 2). Subsequent to treatment discontinuation, 23 (35.4%) patients received additional systemic treatment, with 19 patients receiving a tyrosine kinase inhibitor (14 sorafenib, 3 lenvatinib, 2 cabozantinib), and 4 patients receiving another immune checkpoint inhibitors. There was no difference between patients with and without disease control in terms of gender (*P* = .713), age (< vs ≥65 years; *P* = .531), hepatitis etiology (viral vs non-viral; *P* = .74), presence of cirrhosis (*P* = .543), ECOG-PS (0 vs ≥1; *P* = .972), Child-Pugh classification (A vs B; *P* = .366), median NLR (*P* = .964), or extrahepatic spread (*P* = .74). However, the median AFP level at diagnosis and the frequency of concomitant antibiotic use with immunotherapy were higher in patients without disease control (*P* = .047 and .048, respectively; Table 3).

**Table 2:** Best responses with atezolizumab plus bevacizumab (65 patients).

Response	n	%
Complete response (CR)	1	1.5
Partial response (PR)	23	35.4
Stable disease (SD)	24	36.9
Progressive disease (PD)	17	26.2
Objective response rate (CR + PR)	24	36.9
Disease control rate (CR + PR + SD)	48	73.8

**Table 3:** Comparison of patients with and without disease control.

Parameters	Disease control, n (%)		*P* value
	No, n = 17	Yes, n = 48	
Male sex	15 (88.2)	39 (81.3)	.713
Age ≥ 65 years	7 (41.2)	24 (50)	.531
Viral hepatitis	7 (41.2)	22 (45.8)	.740
Cirrhosis	6 (35.3)	21 (43.8)	.543
ECOG-PS ≥ 1	7 (41.2)	20 (41.7)	.972
AFP, median (range), ng/mL	1000 (1.4–409220)	40.8 (1.8–81658)	**.047**
Child-Pugh class B	3 (17.6)	4 (8.3)	.366
NLR, median (range)	3.09 (0.65–9.12)	3.01 (1.14–18.3)	.964
Extrahepatic spread	10 (58.8)	26 (54.2)	.740
Antibiotic use*	4 (23.5)	3 (6.3)	**.048**

Bold values indicate statistical significance.

AFP = alpha-fetoprotein, ECOG-PS = Eastern Cooperative Oncology Group-Performance Status, NLR = neutrophil-to-lymphocyte ratio.

* Concurrent use of antibiotics with immunotherapy.

### 3.3. Safety profiles

The treatment-related adverse events are summarized in Table 4. Among the study patients, a total of 19 individuals (29.2%) experienced adverse events of any grade, with 7 patients (10.7%) experiencing grade 3 or higher. The most common adverse events of any grade were bleeding events (n = 4, 6.1%), fatigue (n = 3, 4.6%), hypertension (n = 3, 4.6%), diarrhea (n = 3, 4.6%), pruritus (n = 3, 4.6%), and rash (n = 2, 3%). No infusion reactions were reported in any of the patients. Treatment-related toxicity resulted in treatment discontinuation for 7 patients (10.7%), which included 3 cases of bevacizumab-related adverse events (3 bleeding events from gastroesophageal varices) and 4 cases of atezolizumab-related adverse events (1 case each of colitis, nephritis, and hypophysitis).

**Table 4:** Adverse effects of atezolizumab and bevacizumab (65 patients).

Adverse effects		Any grade, n (%)	≥ Grade 3, n (%)
Adverse effects from any cause		19 (29.2)	7 (10.7)
IRAE		9 (13.8)	3 (4.6)
Discontinuation of experimental drug	Atezolizumab and Bevacizumab	–	4 (6.1)
	Bevacizumab only	–	3 (4.6)
Bleeding-related events caused by bevacizumab		3 (4.6)	1 (1.5)
Diarrhoea		3 (4.6)	–
Hypertension		3 (4.6)	–
Fatigue		3 (4.6)	–
Pruritus		3 (4.6)	–
Rash		2 (3)	–
Proteinuria		2 (3)	1 (1.5)
Platelet count decline		2 (3)	–
Aspartate aminotransferase increase		1 (1.5)	–
Immune thyroiditis		7 (10.7)	–
Immune colitis		1 (1.5)	1
Hypophysitis		–	1 (1.5)
Nephritis		1 (1.5)	1 (1.5)

Bold values indicate statistical significance.

IRAE = immune related adverse event.

### 3.4. Survival analysis

**Figure 1.**
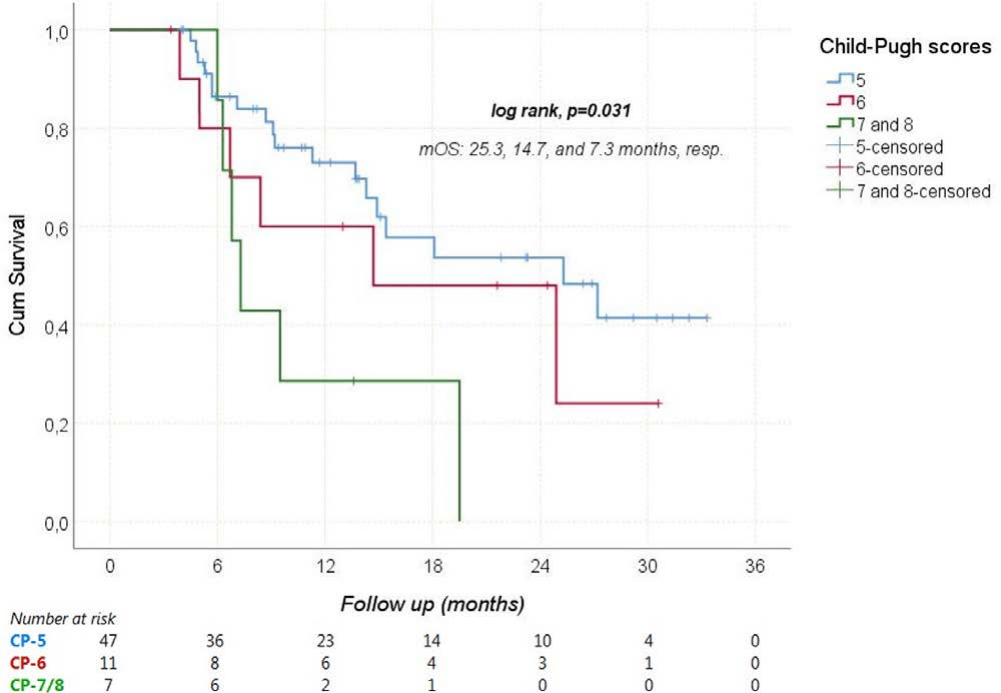
Overal survival according to Child Pugh scores. mOS = median overall survival.

**Figure 2.**
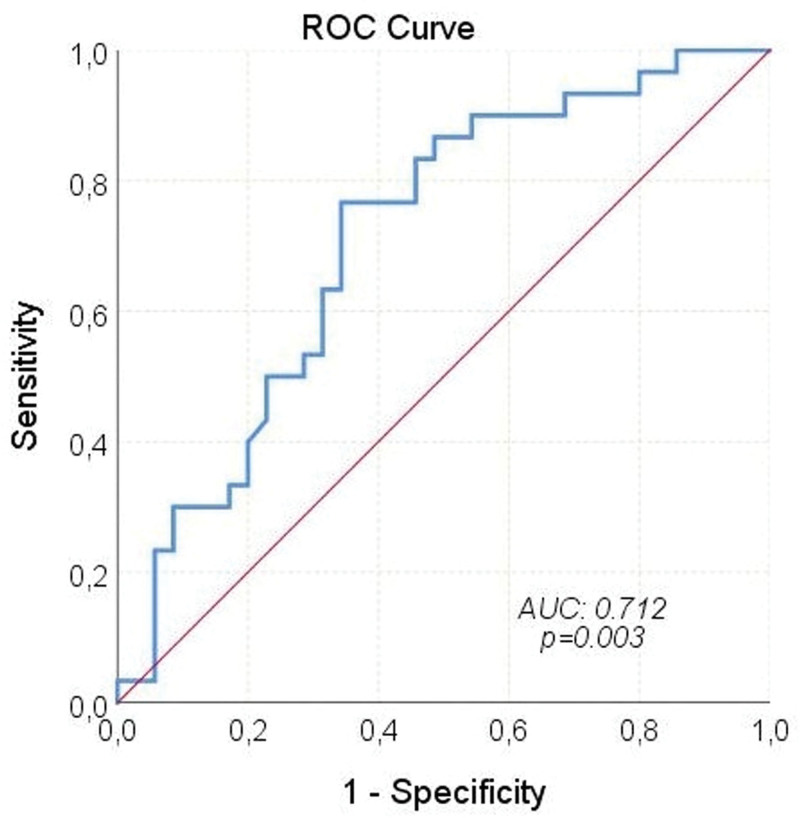
Mortality predictive property of NLR – ROC curve. AUC = area under the curve, NLR = neutrophil-to-lymphocyte ratio.

**Figure 3.**
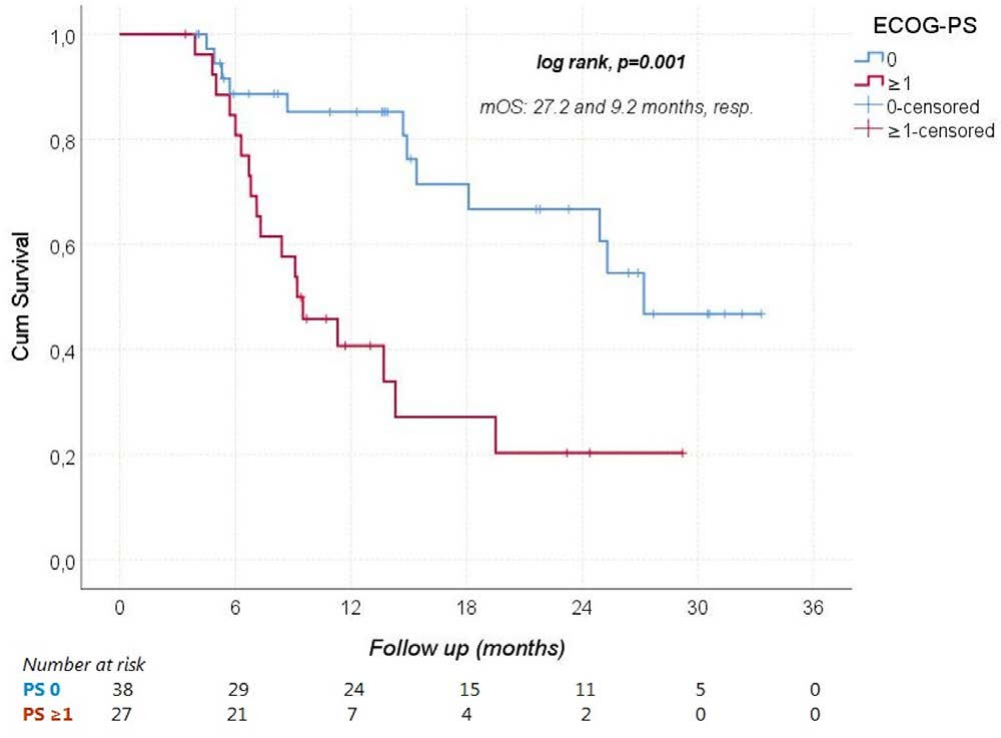
Overal survival according to ECOG-PS. ECOG-PS = Eastern Cooperative Oncology Group-Performance Status, mOS = median overall survival.

**Figure 4.**
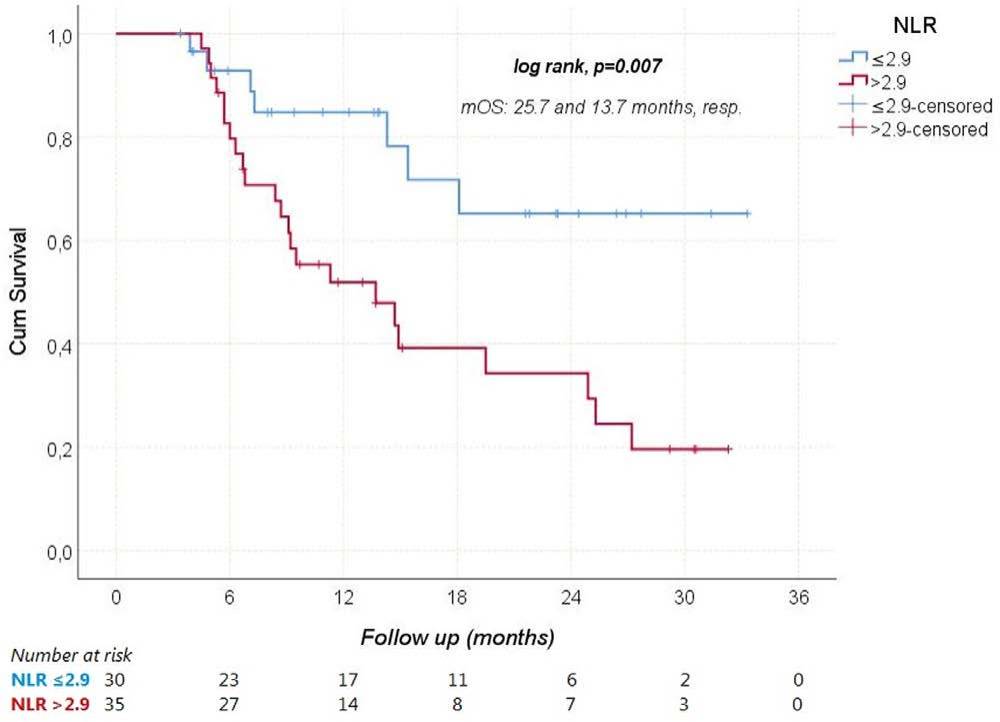
Overal survival according to NLR cutoff. mOS = median overall survival, NLR = neutrophil-to-lymphocyte ratio.

**Figure 5.**
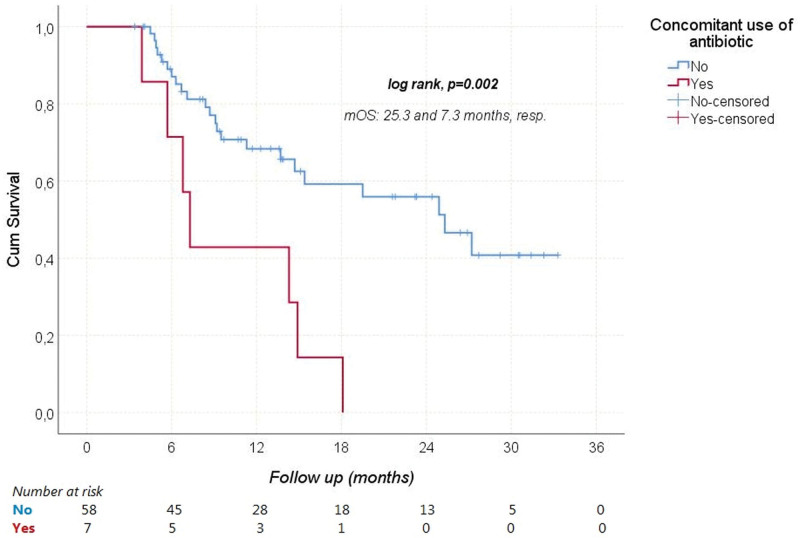
Overal survival according to presence of concomitant antibiotic use. mOS = median overall survival.

During a median follow-up of 11.3 (3.4–33.3) months, HCC progressed in 41 (63.1%) patients and 30 (46.2%) patients died from it. The median PFS and OS were 5.1 (95% CI: 3–7.3), and 18.1 (95% CI: 6.2–29.9) months, respectively. The median OS times according to Child-Pugh scores are shown in Figure 1. Receiver operating characteristic analysis revealed that NLR was a significant predictor of mortality (Area under the curve: 0.712; 95% CI: 0.586–0.838; *P* = .003; Fig. 2). When the cutoff value was > 2.9, the sensitivity and specificity of the test were 76.7% and 65.7%, respectively. In univariate analyses, gender, age ≥ 65 years, viral etiology, AFP ≥ 200 ng/mL, and extrahepatic spread were not found to have any effect on OS. However, ECOG-PS ≥ 1 (relative to 0), Child-Pugh class B (relative to A), NLR > 2.9 (relative to ≤ 2.9), and concomitant antibiotic use with immunotherapy significantly increased the overall risk of mortality (Figs. 3–5). Multivariate analysis revealed that ECOG-PS ≥ 1 (relative to 0; HR: 2.69, *P* = .02), NLR > 2.9 (relative to ≤ 2.9; HR: 2.94, *P* = .017), and concomitant antibiotic use (HR: 4.18, *P* = .003) were independent predictors of OS (Table 5).

**Table 5:** Univariate and multivariate Cox regression analysis of the associations between patient characteristics and overall survival.

Risk factors	Univariate analysis			Multivariate analysis		
	HR	95% CI	*P* value	HR	95% CI	*P* value
Male sex	1.38	0.47–3.99	.551	–	–	–
Age ≥ 65 years	0.94	0.46–1.92	.861	–	–	–
Viral hepatitis	0.91	0.51–2.15	.912	–	–	–
ECOG-PS ≥ 1	3.54	1.65–7.61	**.001**	2.69	1.16–6.20	**.020**
AFP ≥ 200 ng/mL	1.57	0.74–3.32	.236	–	–	–
Child-Pugh class B	3.04	1.21–7.62	**.018**	1.28	0.47–3.53	.628
NLR > 2.9	3.04	1.30–7.12	**.010**	2.94	1.21–7.15	**.017**
Extrahepatic spread	1.50	0.72–3.13	.279	–	–	–
Antibiotic use*	3.64	1.52–8.7	**.004**	4.18	1.65–10.57	**.003**

Bold values indicate statistical significance.

AFP = alpha-fetoprotein, CI = confidence interval, ECOG-PS = Eastern Cooperative Oncology Group-Performance Status, HR = hazard ratio, NLR = neutrophil-to-lymphocyte ratio.

* Concurrent use of antibiotics with immunotherapy.

## 4. Discussion

The combination therapy of Atez/Bev has revolutionized the treatment landscape for HCC, establishing a new standard of care and demonstrating superior long-term efficacy compared to other options.^[16]^ However, the real-world utilization and outcomes of this therapeutic approach have yet to be extensively studied beyond the realm of clinical trials. The primary purpose of this multicenter trial was to assess the safety and efficacy of Atez/Bev when used in ordinary clinical practice. By examining the prospectively maintained global registries of patients treated with immunotherapy, we have confirmed that the combination of Atez/Bev is a safe and effective option when implemented in a clinical setting.^[17–19]^ Here, we present our initial clinical experience with Atez/Bev in HCC patients within a Turkish cohort.

Several real-world studies have investigated the clinical effectiveness and safety of this novel standard treatment.^[20–22]^ These studies have examined the efficacy of the combination therapy in patients who may not have met the original eligibility criteria of the IMbrave150 trial. In the IMbrave150 trial, which included treatment-naïve patients, the ORR was reported as 27.3% and the DCR as 73.6%. The ORR and DCR rates in our study were 36.9% and 73.8%, respectively. The median PFS of 5.1 months in our study was shorter than the reported duration of 6.8 months in the pivotal phase 3 trial. Similarly, the median OS in our study (18.1 months) was slightly shorter than the reported in the IMbrave150 trial (19.2 months). Another study conducted in Germany and Austria, involving 147 HCC patients treated with Atez/Bev, reported outcomes that were inconsistent with the pivotal study. Notably, this study included patients treated in both primary and secondary care settings.^[23]^ The discrepancy in survival outcomes between our study and the IMbrave150 trial may be attributed to the inclusion of patients with higher Child-Pugh scores (7 and 8) and BCLC stage C patients in our cohort. However, we observed a favorable median OS of 25.3 months in patients with a Child-Pugh score of 5. In terms of patient age, the phase 3 IMbrave150 study enrolled 336 patients who received Atez/Bev, with a median age of 64 years (range, 56–71).^[21]^ In our study, the median age of patients was 65 years (range, 22–89), indicating that our cohort included both older and younger patients, unlike the phase 3 study. These real-life cohort data support the use of Atez/Bev therapy in patients of varying ages, including those older and younger than the participants in the pivotal trial.

In terms of OS, our study found that patients with and without viral hepatitis had comparable prognosis. However, an exploratory subgroup analysis of the IMbrave150 trial suggested that patients with viral hepatitis may still benefit from immunotherapy. In addition, Ding et al^[24]^ found no significant difference in ORR between patients with and without virally infected HCC in their meta-analysis (OR: 1.03, 95% CI: 0.77–1.37). Furthermore, our study revealed that patients with NLR ≤ 2.9 had significantly longer OS compared to other groups. This finding aligns with a retrospective study by Eso et al^[25]^ where patients with an NLR score above 3.21 had significantly worse OS (*P* < .001). Regarding antibiotic usage, our study demonstrated that survival rates were significantly higher among patients who did not receive antibiotics during treatment. The utilization of antibiotics was identified as a factor negatively impacting both survival and the effectiveness of immunotherapy. A meta-analysis also indicated that the use of antibiotics during immunotherapy for cancer treatment could significantly shorten PFS and OS while reducing the efficacy of the treatment.^[26]^ However, there is currently no data in the literature specifically assessing the effects of concurrent usage of Atez/Bev and antibiotics on survival and treatment response in advanced HCC patients.

We conducted a comprehensive documentation of all treatment-related adverse events in our study. The combination therapy of Atez/Bev demonstrated favorable tolerability profiles. When comparing our findings to those reported in clinical trials, we did not observe any instances of treatment-related mortality or the emergence of new safety concerns. Specifically, the rates of treatment discontinuation due to treatment-related adverse events were notably similar between our study (6.1%) and the IMbrave150 trial (7%). Regarding safety, the IMbrave150 study reported that adverse events of grade ≥ 3 associated with Atez/Bev therapy were more frequent in the sorafenib group (46%, 71/156) compared to the Atez/Bev therapy group (36%, 117/329). Common adverse events with a rate ≥ 10% in the sorafenib therapy group, such as decreased appetite, hand-foot skin reaction, hypertension, and diarrhea, were less frequent in the Atez/Bev therapy group. In our study, adverse events of any grade occurred in 29.2% of patients. The occurrence of grade ≥ 3 adverse events in our study was 10.7%, which differed from the findings of the IMbrave150 trial, where 38% of patients experienced grade 3 or higher events. It is worth noting that adverse events appear to occur at a significantly lower frequency in our study population, which may indicate underdiagnosis in real-world clinical practice.

This study has several limitations that need to be acknowledged. Firstly, the retrospective design and relatively short follow-up period limit our ability to draw definitive conclusions. Additionally, the real-world nature of our study introduces inherent variability in clinical practices, including differences in eligibility assessment, frequency of follow-up, and management of adverse events. Despite these acknowledged limitations, our study provides further evidence supporting the tolerability and efficacy of the Atez/Bev combination in the management of unresectable HCC in real-world clinical settings.

In summary, the use of atezolizumab plus bevacizumab as a first-line treatment for unresectable HCC has demonstrated comparable effectiveness and safety profiles in real-world settings. However, further extensive investigations with larger sample sizes and longer observation periods are necessary to validate these findings. It is worth noting that while patients with Child-Pugh class B experienced lower OS compared to those with class A, the combination therapy of Atez/Bev demonstrated similar tolerability in Child-Pugh class B patients.

## 5. Conclusion

The combination of atezolizumab and bevacizumab has been established as a safe and effective first-line treatment for patients diagnosed with advanced-stage HCC. Encouragingly, patients with a ECOG-PS score of 0, Child-Pugh score of 5, lower NLR levels, and no history of antibiotic exposure during the treatment demonstrated the greatest potential for achieving enhanced survival outcomes.

## Author contributions

**Conceptualization:** Arif Akyildiz, Deniz Can Guven, Rashad Ismayilov, Omer Dizdar, Erdem Goker, Suayib Yalcin.

**Data curation:** Arif Akyildiz, Deniz Can Guven, Omer Dizdar.

**Formal analysis:** Deniz Can Guven.

**Funding acquisition:** Deniz Can Guven, Ahmet Anil Ozluk, Yakup Iriagac.

**Investigation:** Ahmet Anil Ozluk, Yakup Iriagac, Mert Erciyestepe.

**Methodology:** Arif Akyildiz, Yakup Iriagac, Mert Erciyestepe, Seda Kahraman, Kaan Helvaci, Umut Demirci, Suayib Yalcin.

**Project administration:** Suayib Yalcin.

**Resources:** Emel Mutlu, Olcun Umit Unal, Ibrahim Yildiz, Serdar Turhal, Sinem Akbas, Ertugrul Bayram, Tugba Akin Telli, Fatma Paksoy Turkoz, Melike Ozcelik, Oguzhan Selvi, Burcu Gulbagci, Ismail Erturk, Zehra Sucuoglu Isleyen, Mutianur Ozkorkmaz Akdag, Buket Hamitoglu, Ilkay Tugba Unek, Caglar Unal, İlhan Hacibekiroglu, Cagatay Arslan, Abdulmunir Azizy.

**Software:** Olcun Umit Unal, Ibrahim Yildiz, Sinem Akbas, Tugba Akin Telli, Caglar Unal.

**Supervision:** Sinem Akbas, Caglar Unal, Omer Dizdar, Mert Basaran, Erdem Goker, Mehmet Ali Sendur, Suayib Yalcin.

**Validation:** Melike Ozcelik, Umut Demirci, Mert Basaran, Mehmet Ali Sendur.

**Visualization:** Rashad Ismayilov.

**Writing – original draft:** Arif Akyildiz, Rashad Ismayilov.

**Writing – review & editing:** Deniz Can Guven, Umut Demirci, Omer Dizdar, Mert Basaran, Erdem Goker, Mehmet Ali Sendur, Suayib Yalcin.
